# Ozonation of Popcorn Kernels: Saturation Kinetics at Different Specific Flow Rates, Control of *Aspergillus flavus* Infection, and Grain Quality Analysis

**DOI:** 10.3390/foods13203301

**Published:** 2024-10-17

**Authors:** Marcus Vinícius Assis Silva, Lêda Rita D’Antonino Faroni, Ernandes Rodrigues de Alencar, José Marcelo Soriano Viana, Eugénio da Piedade Edmundo Sitoe, Davi Vittorazzi Salvador, Vivaldo Mason Filho, Carollayne Gonçalves Magalhães

**Affiliations:** 1 Department of Agricultural Engineering, Universidade Federal de Viçosa, Viçosa 36570-900, MG, Brazil; marcus.assis@ufv.br (M.V.A.S.); ernandes.alencar@ufv.br (E.R.d.A.); eugenio.sitoe@ufv.br (E.d.P.E.S.); davi.salvador@ufv.br (D.V.S.); carollayne.magalhaes@ufv.br (C.G.M.); 2 Departament of General Biology, Universidade Federal de Viçosa, Viçosa 36570-900, MG, Brazil; jmsviana@ufv.br; 3 myOZONE Ozone Equipment Industry Eireli, Jaguariúna 13915-294, SP, Brazil; vivaldo@myozone.com.br

**Keywords:** fungal decontamination, ozonation, *Zea mays everta*

## Abstract

Ozone gas (O_3_) is a promising alternative for fungal inactivation in agricultural commodities. This study aimed to (i) investigate the influence of airflow on the saturation of popcorn kernels with ozone gas, (ii) evaluate its effectiveness in controlling *Aspergillus flavus*, and (iii) analyze the quality of ozonated grains. Samples of 3.0 kg of kernels were exposed to oxygen (control) or ozone at specific flow rates of 0.15 or 1.00 m^3^ min^−1^ t^−1^, with an input ozone concentration of 16.0 mg L^−1^ for 0, 6, 12, 24, 36, or 48 h. Quality parameters assessed included expansion volume, water content, electrical conductivity, and color. At 0.15 m^3^ min^−1^ t^−1^, ozone consumption and saturation time were lower, with an 80% reduction in *A. flavus* infection after 6 h. This flow rate did not affect grain expansion or water content. Conversely, at 1.0 m^3^ min^−1^ t^−1^, reductions in water content and expansion were observed with extended exposure. Electrical conductivity increased in both treatments, more significantly at the lower flow rate. In conclusion, ozonation at 0.15 m^3^ min^−1^ t^−1^ effectively inactivated *A. flavus* without compromising grain quality.

## 1. Introduction

Fungal activity on cereal and legume grains can affect their nutritional quality, cause mass loss, and induce organoleptic changes [1]. In seeds, fungi can hinder their germination capacity and viability. In addition to product contamination, certain species of fungi can produce mycotoxins given the proper environmental conditions [2].

Mycotoxins are toxic secondary metabolites with low molecular weight produced by filamentous fungi infecting grains [3]. Grain contamination with these substances can occur in the field and during processing and storage [4]. Fungi from the genera *Aspergillus*, *Penicillium*, and *Fusarium* are the main culprits for the decay of corn grains and their contamination with mycotoxins [5]. Overall, mycotoxins represent great concern, as they pose severe risks to human health [6].

The species *Aspergillus flavus* and *Aspergillus parasiticus* are the main producers of aflatoxins in food [7]. These substances constitute a group of mycotoxins whose most common types are B1 (AFB1), B2 (AFB2), G1 (AFG1), and G2 (AFG2) [8]. They pose a global problem regarding food contamination due to their high carcinogenic, teratogenic, and mutagenic potential, also causing significant economic loss, as contaminated products often need to be discarded [9]. On this account, government agencies in many countries have set maximum tolerated limits (MTLs) for aflatoxins to protect consumers’ health and ensure the purchase of products with high nutritional quality that are free from contaminants [10,11,12].

Popcorn kernels, just like regular corn grains and other cereals, are susceptible to fungal activity and mycotoxin contamination [13,14]. Alborch et al. [13] found mycotoxins in corn flour and popcorn kernels available in Spanish supermarkets. Among 30 samples of popcorn kernels, aflatoxin B1 was detected in 2 of them at a concentration of 3.72 µg kg^−1^, and ochratoxin was identified in 10 samples at levels ranging from 0.79 to 1.71 µg kg^−1^.

In light of the potential harm to consumers’ health and grains, exploring and devising methods for preventing and controlling fungi in food, such as popcorn kernels, is crucial [15]. In this context, ozone gas has emerged as an alternative technology for preserving and decontaminating vegetables, fruits, and dry foods [16].

Ozone is a triatomic gas resulting from the rearrangement of oxygen atoms. It can be artificially produced by electrical discharges or by the incidence of high-energy electromagnetic radiation (ultraviolet light) in the air [17]. The use of ozone gas proved effective in inactivating fungi and breaking down mycotoxins in different agricultural products, such as corn [5,18], peanuts [19], rice [20] and chestnuts [21]. By extension, ozone could potentially be used as an alternative to inactivate aflatoxigenic fungal species in popcorn kernels, thereby preventing the synthesis of aflatoxins. Scientific investigations have already demonstrated that ozone gas applied via forced convection can propagate through large volumes of grains to control insect pests efficiently [22,23,24].

This research investigated different specific flow rates commonly used in low-temperature grain aeration and drying operations for applying ozone gas to popcorn kernels to promote fungal decontamination. The objectives of this study were the following: (i) to determine the influence of airflow on the saturation of popcorn kernels with ozone gas; (ii) to assess the effectiveness of ozone gas in controlling *A. flavus*; and (iii) to characterize the quality of popcorn kernels following ozone exposure.

## 2. Materials and Methods

### 2.1. Popcorn Kernel Samples

Samples of popcorn kernels were purchased at a local supermarket. They were homogenized and kept in a cold room at 5 °C prior to the experiment. The initial characterization of the popcorn kernels was as follows: water content—12.3 ± 0.1 g 100 g^−1^ (wet basis, w.b.); expansion volume—38.5 ± 1 mL g^−1^; percentage of unpopped kernels—1.6 ± 0.6%; flake volume—7.1 ± 0.2 mL; and electrical conductivity—13.9 µS cm^−1^ g^−1^. As for the kernels’ color, the initial values for luminosity (L*), color hue (h*), and color saturation (C*) were 59.55 ± 5.38, 81.13 ± 2.57, and 24.93 ± 4.69. In popped kernels (flakes), L*, h*, and C* were equal to 61.19 ± 7.01, 100.26 ± 1.70, and 7.57 ± 0.99, respectively.

### 2.2. Experimental Procedure

#### 2.2.1. Procedure Prototype

**Figure 1:**
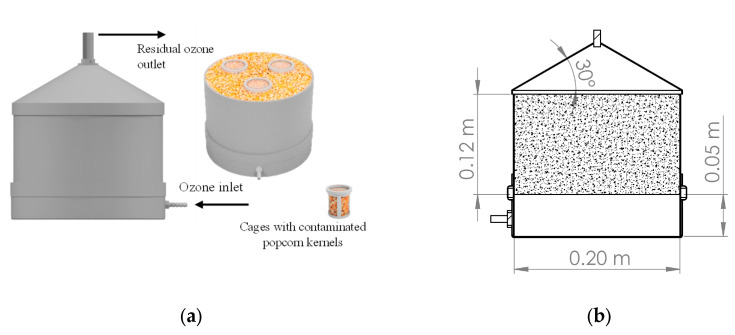
Prototype for popcorn kernel treatment with ozone gas displaying the arrangement of the containers with *A. flavus*-contaminated kernels within the grain mass (**a**). Longitudinal view with dimensions; the hatched area corresponds to the grain mass (**b**).

The experiment’s prototype consisted of a cylindrical PVC column with a metal screen (*plenum*) placed 0.05 m from the bottom. This structure provided support to the grains and facilitated the dispersion of ozone gas throughout the grain mass (Figure 1). The cylindrical column was designed to hold 3.0 kg of popcorn kernels, with dimensions adhering to the height-to-diameter ratio (1.0 to 0.4) recommended by the European standard for low-silo structural design [25].

The prototype was built to avoid the wall effect [26,27], which is a phenomenon that occurs when fluids flow through porous media in a fixed bed. The wall effect indicates a preferential flow along the walls of the device containing the porous medium. This phenomenon must be considered when designing prototypes for small-scale laboratory experiments in order to make the study more representative. The wall effect is eliminated when the ratio between the column (Dc) and particle (D_P_) diameters is respected. This ratio was proposed by Cohen et al. [27], and it should be over 30 for Newtonian fluids and above 50 for non-Newtonian fluids.

Equation (1) was used to obtain the particle diameter (D_P_) for dimensioning the prototype, as described by Karababa [28]. By considering the appropriate height and diameter ratio for designing silos and eliminating the wall effect in the prototype, the objective was to obtain more representative and consistent results for the application of gas into larger volume structures.

(1) $$D_{P}=a\;\text{×}\;{\mathrm{WC}}_{w.b.}+b$$

where

$D_{P}$—average diameter of the particle (mm);
${\mathrm{WC}}_{w.b.}$—water content of the popcorn kernels (on a wet basis);
a and b—constants of the model.

#### 2.2.2. Popcorn Kernel Treatment with Ozone Gas

Ozone was produced using a corona discharge generator model M10 (myOZONE, Jaguariúna, São Paulo, Brazil). A generator model EverFlo (Philips Respironics, Murrysville, PA, USA) supplied the device with oxygen at volumetric flow rates of 0.45 and 3 L min^−1^. The oxygen volumetric flow rate was monitored using a flow meter model MF5700 (Siargo Ltd., Chengdu, China), and the ozone concentration was measured via the iodometric method [29].

Ozone was applied to the 3 kg grain mass at a specific flow rate of 0.15 or 1.00 m^3^ min^−1^ t^−1^, with a corresponding volumetric flow rate of 0.5 or 3 L min^−1^, respectively. These values fall within the recommended limits for operations of aeration (0.1 to 0.5 m^3^ min^−1^ t^−1^) [30,31,32] and drying aeration (0.9 to 2.5 m^3^ min^−1^ t^−1^) [33]. The two specific ozone gas flow rates were tested to determine which was most effective in controlling *A. flavus* while also being replicable for larger product volumes. The control treatment involved applying oxygen at the same specific flow rates used for ozone gas. The input ozone concentration was 16.0 mg L^−1^, and the kernels were exposed to the gas at the aforementioned specific flow rates for 6, 12, 24, 36, or 48 h. The ozone concentration in this study was determined based on preliminary tests and prior research that employed the gas for fungal decontamination in corn grains [5]. The ozone doses corresponding to the specific flow rates of 0.15 and 1.00 m^3^ min^−1^ t^−1^ were 144 and 960 mg h^−1^ kg^−1^, respectively [34].

#### 2.2.3. Saturation of Popcorn Kernels with Ozone Gas

In order to determine the saturation time, the residual ozone concentration was measured after the gas passed through the popcorn kernels. The saturation time was set as the point when the residual ozone concentration stopped varying. The residual ozone concentration data as a function of time were adjusted to a sigmoidal equation (Equation (2)). After fitting it to the data, the saturation time was obtained from Equation (3), as proposed by Venegas et al. [35]. With knowledge of the volumetric flow rate, the cross-sectional area of the flow, and the height of the grain layer, it was possible to use Equation (4) to determine the residence time of ozone gas in the system [23]. The ozone mass injected into the grain mass was calculated ($C_{O_{3}}$) considering the volumetric flow rate, the gas inlet concentration, and the exposure time (Equation (5)). The ozone consumption by the grain mass was determined based on an adjusted equation describing the residual ozone concentration as a function of time (Equation (6)).

(2) $$C=\frac{a}{\left(1+e^{-\left(\frac{t\;-\;b}{c}\right)}\right)}$$

(3) $$t_{\mathrm{sat}}=b+2c$$

(4) $$t_{\mathrm{res}}=\frac{\text{ɛ}}{Q}\;\text{×}\;A\;\text{×}\;H$$

(5) $$C_{O_{3}}=\int_{0}^{t}C_{0}\mathrm{Qdt}$$

(6) $$C_{O_{3}\mathrm{grains}}=\int_{0}^{t}C_{0}\mathrm{Qdt}-\int_{0}^{t}\frac{a}{1+e^{-\left(\frac{t\;-\;b}{c}\right)}}\mathrm{Qdt}$$

where

C—ozone gas concentration (mg L^−1^);
t—time (min);
Q—ozone gas volumetric flow rate (L min^−1^);
A—airflow cross-section (m^2^);
H—grain layer height (m);
ɛ—grain porosity;
$C_{O_{3}}$—total ozone mass injected into the grain mass (g);
$C_{O_{3}\mathrm{grains}}$—ozone consumed by the grain mass (g);
a, b, and c—constants;
t_sat_—saturation time (h);
t_res_—residence time (min).

### 2.3. Control of A. flavus in Popcorn Kernels Treated with Ozone

#### 2.3.1. Inoculation of *A. flavus* into Popcorn Kernels

*A. flavus* CCUB1405 was obtained from symptomatic commercial peanuts using *A. flavus*-*parasiticus* agar (AFPA) as the culture medium [36] (p. 519). This strain was identified through sequence analysis of the ITS and partial beta-tubulin gene (BenA) [21]. A sequence of this isolate was deposited in the GenBank database under the access number CCUB1405.

*A. flavus* CCUB1405 was inoculated into popcorn kernels by adding 10 mL of sterile distilled water to each culture plate, and the resulting conidium suspension was adjusted to 10^5^ conidia mL^−1^. The popcorn kernels were immersed in this suspension for 10 min. After this, a 2 cm thick layer of the sample was placed on a flat surface and kept at room temperature for seven days. This period was necessary to naturally remove the surface water of the grains.

#### 2.3.2. *A. flavus* Count

Determining the ozone potential in inactivating *A. flavus* involved inserting three containers with 30.0 g of fungus-inoculated popcorn kernels into the grain mass (Figure 1a). The containers consisted of a PVC frame covered with organza-type fabric (100% polyester, 35 g m^−2^, Eur/Fabtex), which held the grains while allowing the axial and radial ozone flow. After exposure to ozone for 6, 12, 24, 36, or 48 h, the grains were removed from the containers and directly plated on *Aspergillus flavus-parasiticus* agar (AFPA) to assess the percentage of infection by *A. flavus* [36]. Fifteen grains were used per plate, either with or without surface disinfection—a sodium hypochlorite solution at 0.4 g 100 mL^−1^ was used for surface disinfection. The plates were incubated at 30 °C for 42 h [37]. Subsequently, the grains showing the development of yellow–orange colonies were counted, and the results were expressed in percentage.

### 2.4. Popcorn Kernel Quality

The quality variables investigated in popcorn kernels exposed to ozone gas or oxygen (control) at two specific flow rates and for five exposure periods were kernel expansion, water content, electrical conductivity, and color. All grain quality analyses were carried out in triplicate, and the mean with standard deviations was calculated.

#### 2.4.1. Popcorn Kernel Expansion

Kernel expansion analysis considered the expansion volume, flake volume, and percentage of unpopped kernels. The expansion volume was determined using the methodology of Mendez et al. [22] and Silva et al. [38]. Grain samples of 250 g were subjected to the expansion process using 100 mL of coconut oil in a metric weight volume tester (MWVT) model CR13101 (Cretors & Company, Chicago, IL, USA). This device consists of a batch-type popcorn maker that pops the kernels evenly, collecting the flakes in a calibrated cylinder to determine the expansion volume in mL g^−1^. Flake volume and the percentage of unpopped kernels were determined based on the average number of grains in the 2250 g samples [39].

#### 2.4.2. Water Content

The water content of the kernels was determined by placing them in an oven with forced air circulation at 103 °C for 72 h, following the standard method described by the ASAE (American Society of Agricultural Engineers) [40]. The results were expressed on a wet basis (g 100 g^−1^ w.b.).

#### 2.4.3. Electrical Conductivity

Electrical conductivity was analyzed using the methodology proposed by Vieira et al. [41]. For each combination of specific flow rate and exposure period to ozone or oxygen (control), 50 g samples were placed in plastic containers filled with 75 mL of distilled water. The samples were stored in a biochemical oxygen demand chamber at 25 °C for 24 h. After that, the electrical conductivity of the immersion solution was read with a conductivity meter model MCA-150 (Tecnopon, Piracicaba, São Paulo, Brazil), and the results were expressed in µS cm^−1^ g^−1^.

#### 2.4.4. Color

Color evaluation of the popcorn kernels and flakes involved measuring the direct reflectance of the coordinates L* (white-to-black intensity), a* (red-to-green intensity), and b* (yellow-to-blue intensity), using a colorimeter model CR410 (Konica Minolta, Osaka, Japan). The values of L*, a*, and b* allow for the determination of the color hue or hue angle (h*, Equation (5)), color saturation or chroma (C*, Equation (6)), and total color difference (∆E*, Equation (7)). The coordinates L*, a*, and b* of samples not exposed to ozone or oxygen were used as reference to calculate the total color difference.

(7) $$C^{*}=\sqrt{{a*}^{2}+{b*}^{2}}$$

(8) $$C^{*}=\sqrt{{a*}^{2}+{b*}^{2}}$$

(9) $${\text{∆}E}^{*}=\sqrt{{\left(L^{*}-L_{0}^{*}\right)}^{2}+{\left(a^{*}-{\;a}_{0}^{*}\right)}^{2}+{\left(b^{*}-b_{0}^{*}\right)}^{2}}$$

where $L_{0}^{*}$, $a_{0}^{*}$, and $b_{0}^{*}$ correspond to the coordinates before kernel exposure to ozone or oxygen.

### 2.5. Statistical Analysis

The residual ozone concentration data for the two specific flow rates and the quality variables as a function of time were submitted for regression analysis. The models were chosen based on the significance of the regression coefficients, as determined by the *t*-test at a 5% probability level for both the coefficient of determination (*R^2^*) and the phenomenon under study. The statistical analysis and graph plotting were handled by the software SigmaPlot version 12.0 (Systat Software Inc., Erkrath, Germany) [42].

## 3. Results

### 3.1. Saturation of Popcorn Kernels with Ozone Gas

**Figure 2:**
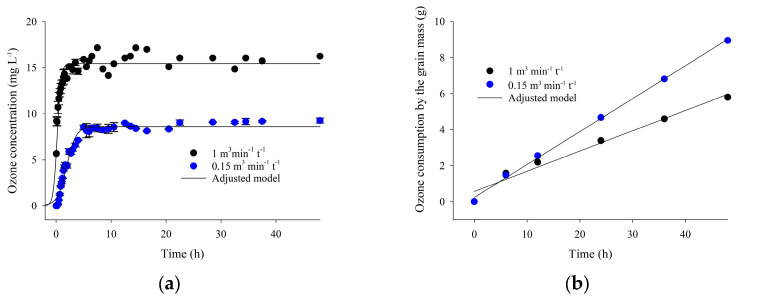
Residual ozone gas concentration as a function of time during the saturation of popcorn kernels (**a**) and ozone consumption by the grain mass (**b**), considering an input concentration of 16 mg L^−1^ and specific flow rates of 0.15 and 1.00 m^3^ min^−1^ t^−1^.

Figure 2a depicts the variation in residual ozone concentration as a function of time during the saturation of popcorn kernels with ozone at 16 mg L^−1^ and a specific flow rate of 0.15 or 1.00 m^3^ min^−1^ t^−1^. Figure 2b exhibits the relationship between ozone consumption by the grain mass and exposure duration. The adjusted equations describing the variation in residual ozone concentration during the saturation process are presented in Table 1. Table 2 contains the adjusted equations that explain the ozone consumption by the popcorn kernel mass as a function of the exposure period. At a specific flow rate of 0.15 m^3^ min^−1^ t^−1^, the total mass of ozone injected into the grains, residence time, saturation time, and saturation concentration were 18.43 g, 6.72 min, 3.59 h, and 8.59 mg L^−1^, respectively (Table 1). At 1.00 m^3^ min^−1^ t^−1^, these values were equal to 138.24 g, 1.12 min, 0.88 h, and 15.44 mg L^−1^ (Table 1).

### 3.2. Control of A. flavus in Popcorn Kernels with Ozone

**Figure 3:**
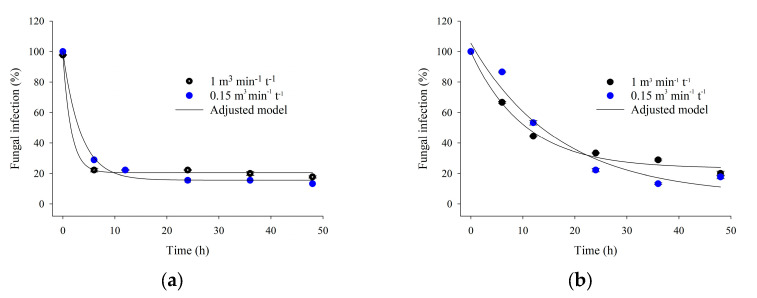
Percentage of *A. flavus* infection in popcorn kernels as a function of the exposure duration to ozone gas at different specific flow rates, considering grains with surface disinfection (**a**) and grains without surface disinfection (**b**).

**Figure 4:**
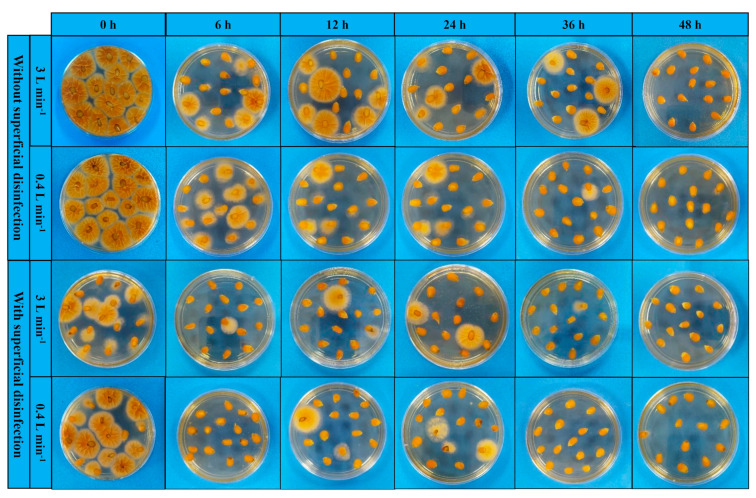
This direct plating of popcorn kernels exposed to ozone or oxygen (control) for different exposure periods and at different flow rates of ozone gas.

Figure 3 shows the percentage of *A. flavus* infection in popcorn kernels as a function of the exposure duration to ozone gas applied at 16 mg L^−1^ and at a specific flow rate of 0.15 or 1.00 m^3^ min^−1^ t^−1^. Figure 3 demonstrates the percentage reduction in *A. flavus* infection in kernel samples using the technique of direct plating without surface disinfection (Figure 3a) and with surface disinfection (Figure 3b). Ozone gas applied at a specific flow rate of 0.15 or 1.00 m^3^ min^−1^ t^−1^ efficiently decreased the percentage of *A. flavus* infection in the grains (Figure 3 and Figure 4). Notably, the internal *A. flavus* infection was reduced by 80% after 6 h of exposure to ozone gas, as demonstrated by direct plating with surface disinfection (Figure 3a). Table 3 shows the regression equations describing the relationships depicted in Figure 3.

### 3.3. Popcorn Kernel Quality

Table 4 presents the adjusted regression equations, along with the respective determination coefficient (*R^2^*) and standard error of estimate (SEE), for the quality analysis of samples exposed to ozone or oxygen gas (control) at a specific flow rate of 0.15 or 1.00 m^3^ min^−1^ t^−1^. The regression equations represent the expansion volume, flake volume, unpopped kernel percentage, water content, electrical conductivity, and color.

#### 3.3.1. Popcorn Kernel Expansion

**Figure 5:**
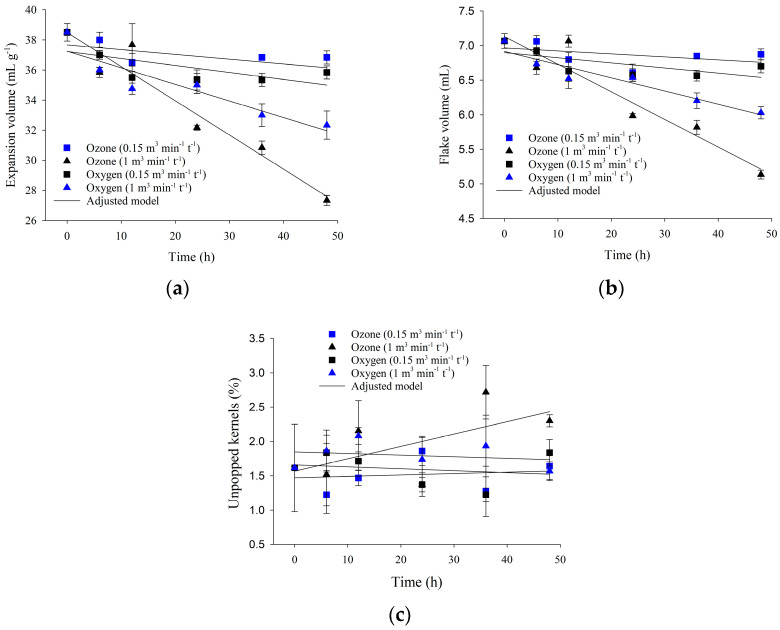
Expansion volume (**a**), flake volume (**b**), and unpopped kernel percentage (**c**) as a function of ozone or oxygen exposure at different exposure periods.

When ozone treatment was applied at a specific flow rate of 0.15 m^3^ min^−1^ t^−1^ for up to 48 h, it did not impact the expansion volume, flake volume, and percentage of unpopped kernels (Table 4; Figure 5). The same trend was verified when only oxygen was used.

On the other hand, oxygen treatment at the specific flow rate of 1.00 m^3^ min^−1^ t^−1^ and ozone treatment at the same flow rate for up to 48 h significantly altered the expansion volume (Table 4; Figure 5a) and flake volume (Table 4; Figure 5b). As for unpopped kernels (Table 4; Figure 5c), there was no significant change (*p* < 0.05) related to the exposure period of the grains to ozone gas or oxygen. As kernel exposure to ozone or oxygen extended, expansion volume and flake size decreased. This reduction was more significant in the treatment with ozone.

#### 3.3.2. Water Content

Regarding water content, the duration of exposure to ozone or oxygen did not significantly affect the kernels when either gas was injected at the specific flow rate of 0.15 m^3^ min^−1^ t^−1^ (Table 4). When ozone was injected at a specific flow rate of 1.00 m^3^ min^−1^ t^−1^, the water content decreased as a function of the exposure period in the treatments with ozone or oxygen (Table 4).

#### 3.3.3. Electrical Conductivity

**Figure 6:**
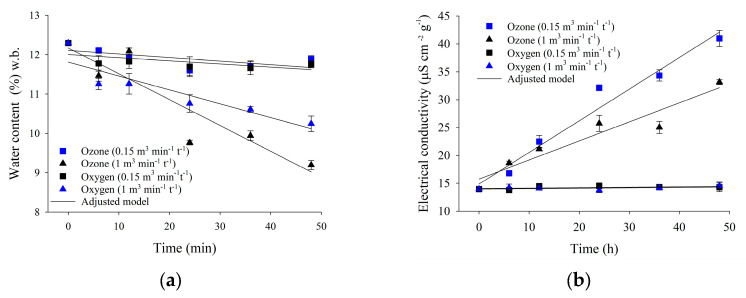
Water content (**a**) and electrical conductivity (**b**) of popcorn kernels as a function of the exposure period to ozone gas.

The electrical conductivity was affected by the ozone gas treatments at both volumetric flow rates (0.15 and 1.00 m^3^ min^−1^ t^−1^). On the other hand, exposure to oxygen at the same specific flow rates did not result in significant changes (Table 4; Figure 6b). Conductivity increased linearly as a function of the exposure period to ozone (Figure 6b). As the exposure periods increased, the samples treated with ozone gas injected at a specific flow rate of 0.15 m^3^ min^−1^ t^−1^ exhibited higher conductivity than those treated with ozone gas at 1.00 m^3^ min^−1^ t^−1^.

#### 3.3.4. Color

**Figure 7:**
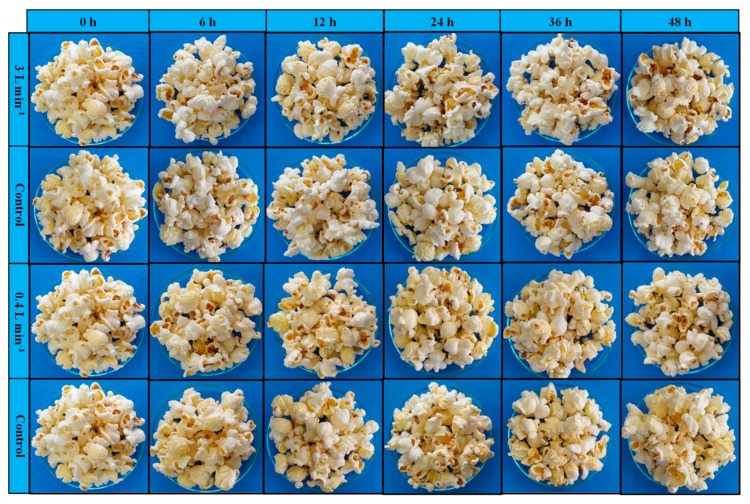
Popcorn flakes exposed to ozone or oxygen for different durations and flow rates.

The exposure duration to ozone gas or oxygen did not change the color tone (h*), color saturation (C*), and color difference (ΔE*) of the popcorn kernels in either specific flow rate tested (0.15 or 1.00 m^3^ min^−1^ t^−1^) (Table 4). As for the popcorn flakes, only color hue (h*) varied as a function of the exposure period to ozone gas, decreasing as the exposure to the gas extended (Table 4). Figure 7 shows the visual aspect of popcorn obtained from grains subjected or not to ozonation under different conditions.

## 4. Discussion

The results for the saturation kinetics of popcorn kernels followed the same pattern as reported in other scientific works that investigated the saturation of grains with ozone gas [18,22,23,43]. According to these studies, ozone flows through a grain mass in two phases regardless of the specific flow rate. At first, the interaction between ozone and the grains is intense, and the gas quickly decomposes into oxygen. In a subsequent stage, once all the active sites of the product have reacted with ozone, the gas moves freely through the grain mass, indicating that the saturation state has been achieved [22].

The first phase of the grain saturation process was more evident when using a specific flow rate of 0.15 m^3^ min^−1^ t^−1^ (Figure 2a). At this particular flow rate, the residence time of ozone with the grains was longer (6.72 min) compared to when it was injected at 1.00 m^3^ min^−1^ t^−1^ (1.12 min). A prolonged residence implies increased contact and higher reaction rates between ozone and the grains, thus favoring gas decomposition [23,24]. This explains why the saturation time at a specific flow rate of 0.15 m^3^ min^−1^ t^−1^ was higher (3.59 h) and the saturation concentration lower (8.59 mg L^−1^) than the results at 1.00 m^3^ min^−1^ t^−1^.

Calculating the total ozone mass injected into the grains and the gas consumption also allowed for observing the influence of different specific flow rates on the saturation process. When adopting a specific flow rate of 1.00 m^3^ min^−1^ t^−1^, the total ozone mass injected was 7.5 times (138.24 g) the value obtained at 0.15 m^3^ min^−1^ t^−1^ (18.43 g). Conversely, ozone consumption by the grain mass exhibited the opposite behavior. A specific flow rate of 0.15 m^3^ min^−1^ t^−1^ resulted in higher ozone consumption by the grain mass compared to the treatment at 1.00 m^3^ min^−1^ t^−1^ (Figure 2a; Table 1). When using higher specific flow rates, the grain mass reaches saturation faster. However, there is a greater oxygen demand to supply ozone production, which implies more significant expenses, especially in ozone generation systems that use industrial oxygen as an input. On the other hand, lower specific flow rates require a longer time to saturate the grain mass and proceed with the treatment.

The decreased percentage of grain infection by *A. flavus* can be attributed to the oxidative potential of ozone gas (2.07 mV) [44,45,46]. Previous research demonstrated that ozone gas efficiently inactivated fungi in peanuts [19,47], wheat [48], rice [20,49], and common corn [5,18].

Molecular ozone and the free radicals generated during its decomposition act on components of the membrane and cell wall of microorganisms, compromising their integrity and leading to the leakage of intracellular content [50,51]. This explains the inactivation of *A. flavus* in popcorn kernels. It is important to note that the specific flow rate did not affect the reduction in the percentage of grain infection by *A. flavus*.

Expansion volume is a quality variable that encompasses flake volume and the percentage of unpopped kernels. Only the popcorn kernels exposed to ozone at a specific flow rate of 1.00 m^3^ min^−1^ t^−1^ exhibited a decreased expansion volume. Flake volume displayed a similar pattern, and the percentage of unpopped kernels remained unaltered, considering the different specific flow rates tested. This is evidence that the reduction in expansion volume was solely due to a decrease in flake volume. This result suggests that adopting lower specific flow rates may have the same effectiveness, allowing for controlling *A. flavus* without incurring unnecessary costs or compromising product quality.

The reduction in flake volume (and thereby expansion volume) is linked to the kernels losing water [52] and potentially sustaining physical damage [53]. Even though the expansion volume also decreased in the control group, the phenomenon was more pronounced in grains treated with ozone. Expansion volumes below 30 mL g^−1^ were observed when adopting a specific flow rate of 1.00 m^3^ min^−1^ t^−1^ and a 48 h exposure. These values are below the limits established by the Brazilian Normative Ruling No. 61/2011 [54], which means the kernels do not meet the marketing requirements. This reduction in expansion volume is due to ozone likely increasing the fragility of the pericarp, thereby compromising the integrity of the grains.

A whole pericarp is the main requirement for proper kernel expansion [55]. Grains with a fragile pericarp may release some internal pressure before popping, resulting in lower expansion volumes. Pohndorf et al. [53] observed this phenomenon when evaluating the influence of temperature and the drying air speed on the expansion properties of red popcorn kernels. According to these authors, cracks may form when the grains undergo temperatures between 70 and 100 °C, and these fissures release some internal pressure before the grain pops, therefore reducing volume yield.

The purpose of aeration is to ensure an even distribution of temperature and relative humidity of the air in the inter-grain space without affecting the water content of the grains. A variation in water content was not expected when a specific flow rate of 0.15 m^3^ min^−1^ t^−1^ was employed, as this value falls within the range recommended for grain aeration [30,31,32]. The reduction in water content verified in the grains treated at 1.00 m^3^ min^−1^ t^−1^ was consistent with conditions typically employed for grain drying [33].

Furthermore, using moisture-free oxygen to generate ozone favored the reduction in the water content of the grains [21]. Mendez et al. [22] also observed a reduction in the water content of popcorn kernels treated with ozone gas in a flow. Such decreases are not exclusively caused by ozone gas but by the specific airflow adopted, as higher specific flow rates imply higher mass transfer coefficients. This can account for the decrease in water content in the grains treated at 1.00 m^3^ min^−1^ t^−1^ with either ozone or oxygen [56,57].

The physical integrity of grains and seeds can be indirectly assessed via electrical conductivity [41,58]. The greater the damage to the grain’s physical integrity, the more ions are released and the higher the electrical conductivity of the immersion solution. Previous research demonstrated that ozone gas affects the electrical conductivity of popcorn kernels [38,43].

In this study, electrical conductivity was found to be highly sensitive to ozone gas treatment, and the longer the exposure to ozone gas, the higher the electrical conductivity (Table 3; Figure 6b). The electrical conductivity of the kernel samples exposed to ozone at a flow rate of 0.15 m^3^ min^−1^ t^−1^ was higher than those exposed at 1.00 m^3^ min^−1^ t^−1^. This can be explained by the fact that lower specific flow rates represent a lower interstitial velocity and, consequently, a more extended ozone residence (Table 1). Despite this, the grains ozonated at a specific flow rate of 0.15 m^3^ min^−1^ t^−1^ showed greater expansion volume than those treated at 1.00 m^3^ min^−1^ t^−1^ (Figure 5).

Longer residence times allow for greater interaction between ozone gas and the grains, as well as higher reaction rates and greater ozone consumption (Figure 2b; Table 1). The graph in Figure 2b depicts the consumption of ozone by the grains. It exhibits the same behavior as the graph in Figure 6b, which compares the electrical conductivity associated with each ozone specific flow rate tested.

The grains subjected to the lower specific flow rate (0.15 m^3^ min^−1^ t^−1^) showed greater ozone consumption and higher electrical conductivity of the immersion solution (Table 2). On the other hand, the higher specific flow rate (1.00 m^3^ min^−1^ t^−1^) led to lower electrical conductivity values and less ozone consumption by the grain mass. Based on the quality analyses regarding popcorn kernel expansion, it could be inferred that the increase in the electrical conductivity values, especially in the treatments at a specific flow rate of 0.15 m^3^ min^−1^ t^−1^, was not responsible for the reduced grain expansion and flake volume. The reduction in water content possibly had a more significant impact on grain expansion than the electrical conductivity.

Color is one of the quality variables of great interest, especially in ready-to-eat foods, such as popcorn [57,59,60]. The color parameters of the popcorn kernels remained unchanged across all treatments. Silva et al. [38] reported similar findings when they assessed the color of popcorn kernels exposed to ozone gas in a low-pressure injection system.

In the present study, the color of popcorn flakes was also evaluated, and no alterations were observed in color saturation (C*) or color difference (ΔE*). However, a reduction in color hue (h*) as a function of the exposure period was observed in the ozone treatment at a specific flow rate of 1.00 m^3^ min^−1^ t^−1^. Previous studies reported that ozone increased luminosity (L*) and decreased yellowing in corn and sorghum flour [61,62].

## 5. Conclusions

The application of ozone at various flow rates affected the saturation process of popcorn kernels with the gas. Injecting ozone at the lower specific flow rate resulted in a slower saturation of the grain mass. When using higher specific flow rates, the process of saturating the grain mass with ozone is faster. However, there is a greater oxygen demand to supply ozone production, which results in a less economical process, especially in ozone generation systems that use industrial oxygen as an input. On the other hand, lower specific flow rates require a longer time to saturate the grain mass and proceed with the treatment. Regarding the reduction in *A. flavus* infection percentage, ozonation was effective at both specific flow rates tested. However, at a specific flow rate of 1.00 m^3^ min^−1^ t^−1^, changes were observed in the expansion, water content, and electrical conductivity of the kernels. Ultimately, this study proved that applying ozone at a specific flow of 0.15 m^3^ min^−1^ t^−1^ effectively inactivates *A. flavus* without affecting the quality of popcorn kernels.

## Figures and Tables

**Table 1:** Regression equations adjusted based on the variation in residual ozone concentration during popcorn kernel saturation, considering an input concentration of 16 mg L^−1^ and specific flow rates of 0.15 and 1.00 m^3^ min^−1^ t^−1^.

Specific Flow Rate (m^3^ min^−1^ t^−1^)	Adjusted Equations	$t_{res}$(min)	$t_{\mathrm{sat}}$ (h)	$C_{\mathrm{sat}}$(mg L^−1^)	$C_{O_{3}}$(g)	*R^2^*	SEE
0.15	$\hat{C}=\frac{8.59}{\left(1+e^{-\left(\frac{t\;-\;1.89}{0.85}\right)}\right)}$	6.72	3.59	8.59	18.43	0.96	0.59
1.0	$\hat{C}=\frac{15.44}{\left(1+{\;e}^{-\left(\frac{t\;-\;0.20}{0.34}\right)}\right)}$	1.12	0.88	15.44	138.24	0.84	1.40

t_sat_—saturation time; $t_{\mathrm{res}}$—ozone residence time; $\hat{C}$—estimated ozone concentration; $C_{O_{3}}$—total mass of ozone injected into the grain mass (g); $C_{\mathrm{sat}}$—saturation concentration; *R^2—^* coefficient of determination; SEE—standard error of estimate.

**Table 2:** Regression equations adjusted based on the relationship between ozone consumption by the grain mass and the grain exposure period.

Specific Flow Rate (m^3^ min^−1^ t^−1^)	Adjusted Equations	*R^2^*	SEE
0.15	${\hat{C}}_{O_{3}\mathrm{grains}\;}=0.23+0.18t$	0.99	0.15
1.0	${\hat{C}}_{O_{3}\mathrm{grains}\;}=0.56+0.11t$	0.97	0.37

*R^2^*—coefficient of determination; SEE—standard error of estimate.

**Table 3:** Adjusted regression equations representing the percentage reduction in *A. flavus* contamination (with or without surface disinfection) in popcorn kernels as a function of exposure duration to ozone gas applied at 16 mg L^−1^ and a specific flow rate of 0.15 or 1.00 m^3^ min^−1^ t^−1^.

Specific Flow Rate (m^3^ min^−1^ t^−1^)	With Surface Disinfection		Without Surface Disinfection	
	Adjusted Equations	*R^2^*	Adjusted Equations	*R^2^*
0.15	$\hat{y}=15.70+84.18e^{(-0.29t)}$	0.99	$\hat{y}=5.42+100.02e^{(-0.06t)}$	0.99
1.0	$\hat{y}=20.53+77.25e^{(-0.63t)}$	0.99	$\hat{y}=23.30+76.55e^{(-0.09t)}$	0.96

$\hat{y}$—estimated percentage of contamination reduction by *A. flavus* in popcorn kernels.

**Table 4:** Adjusted regression equations for expansion volume, water content, and electrical conductivity of popcorn kernels, and color parameters of kernels and flakes (color hue h*, color saturation C*, and color difference ΔE*) as a function of the exposure duration to ozone gas or oxygen applied at 16 mg L^−1^ and at a specific flow rate of 0.15 or 1.00 m^3^ min^−1^ t^−1^.

Specific Flow Rate (m^3^ min^−1^ t^−1^)	Control (O_2_)			Ozone (O_3_)		
	Adjusted Equations	*R^2^*	SEE	Adjusted Equations	*R^2^*	SEE
Popcorn kernel expansion						
Expansion volume						
0.15	$\hat{y}=37.23$	-	-	$\hat{y}=37.66$	-	-
1.0	$\hat{y}=37.24\;-0.11\;\text{**}\;t$	0.85	0.96	$\hat{y}=38.48\;-0.23\;\text{**}\;t$	0.93	1.26
Flake volume						
0.15	$\hat{y}=6.89$	-	-	$\hat{y}=6.96$	-	-
1.0	$\hat{y}=6.91\;-\;0.02\;\text{**}\;t$	0.90	0.12	$\hat{y}=7.13\;-0.04\;\text{**}\;t$	0.90	0.26
Percentage of unpopped kernels						
0.15	$\hat{y}=1.66$	-	-	$\hat{y}=1.47$	-	-
1.0	$\hat{y}=1.84$	-	-	$\hat{y}=1.56$	-	-
Water content						
0.15	$\hat{y}=12.00$	-	-	$\hat{y}=12.11$	-	-
1.0	$\hat{y}=11.81-0.03\;*t$	0.83	0.33	$\hat{y}=12.17\;-0.06\;\text{**}\;t$	0.85	0.57
Electrical conductivity						
0.15	$\hat{y}=13.96$	-	-	$\hat{y}=14.86+\;0.57\;\text{**}\;t$	0.96	2.18
1.0	$\hat{y}=14.04$	-	-	$\hat{y}=15.76+0.34\;\text{**}\;t$	0.91	2.16
Popcorn kernel color						
Color hue (h*)						
0.15	$\hat{y}=80.49$	-	-	$\hat{y}=80.20$	-	-
1.0	$\hat{y}=80.77\;$	-	-	$\hat{y}=80.58$	-	-
Color saturation (C*)						
0.15	$\hat{y}=24.36$	-	-	$\hat{y}=24.60$	-	-
1.0	$\hat{y}=23.06$	-	-	$\hat{y}=23.07\;$	-	-
Color difference (ΔE*)						
0.15	$\hat{y}=4.77$	-	-	$\hat{y}=4.42$	-	-
1.0	$\hat{y}=6.54$	-	-	$\hat{y}=5.64$	-	-

* Significant at a 5% probability level according to the *t*-test (*p* < 0.05). ** Significant at a 1% probability level according to the *t*-test (*p* < 0.05).

## Data Availability

The original contributions presented in the study are included in the article, further inquiries can be directed to the corresponding author.

## References

[1] Alconada T.M., Moure M.C. (2022). Deterioration of lipids in stored wheat grains by environmental conditions and fungal infection-A review. J. Stored Prod. Res..

[2] Mohapatra D., Kumar S., Kotwaliwale N., Singh K.K. (2017). Critical factors responsible for fungi growth in stored food grains and non-chemical approaches for their control. Ind. Crops Prod..

[3] Hussein H.S., Brasel J.M. (2001). Toxicity, metabolism, and impact of mycotoxins on humans and animals. Toxicology.

[4] Neme K., Mohammed A. (2017). Mycotoxin occurrence in grains and the role of postharvest management as a mitigation strategies. A review. Food Control.

[5] Ribeiro D.F., Faroni L.R.D.A., Pimentel M.A.G., Prates L.H.F., Heleno F.F., De Alencar E.R. (2022). Ozone as a fungicidal and detoxifying agent to maize contaminated with fumonisins. Ozone Sci. Eng..

[6] Ruyck K., De Boevre M., Huybrechts I., De Saeger S. (2015). Dietary mycotoxins, co-exposure, and carcinogenesis in humans: Short review. Mutat. Res. Rev. Mutat. Res..

[7] Gizachew D., Chang C.H., Szonyi B., De La Torre S., Ting W.T.E. (2019). Aflatoxin B1 (AFB1) production by *Aspergillus flavus* and *Aspergillus parasiticus* on ground Nyjer seeds: The effect of water activity and temperature. Int. J. Food Microbiol..

[8] Coppock R.W., Christian R.G., Jacobsen B.J. (2018). Aflatoxins. Veterinary Toxicology.

[9] Ismail A., Gonçalves B.L., de Neeff D.V., Ponzilacqua B., Coppa C.F., Hintzsche H., Sajid M., Cruz A.G., Corassin C.H., Oliveira C.A. (2018). Aflatoxin in foodstuffs: Occurrence and recent advances in decontamination. Food Res. Int..

[10] Guidance for Industry: Fumonisin Levels in Human Foods and Animal Feeds (2001). https://www.fda.gov/RegulatoryInformation/Guidances/ucm109231.htm.

[11] EC. European Comission (2010). Commission Regulation n. 165/2010 of 26 February 2010 amending Regulation EC n. 1881/2006 setting maximum levels for certain contaminants in foodstuffs as regards aflatoxins. Off. J. Eur. Union.

[12] BRAZIL (2011). Resolução n° 7, de 18 de Fevereiro de 2011. Dispõe sobre Limites Máximos Tolerados (LMT) Para Micotoxinas em Alimentos. Diário Oficial [da] República Federativa do Brasil. https://bvsms.saude.gov.br/bvs/saudelegis/anvisa/2011/res0007_18_02_2011_rep.html.

[13] Alborch L., Bragulat M.R., Castellá G., Abarca M.L., Cabañes F.J. (2012). Mycobiota and mycotoxin contamination of maize flours and popcorn kernels for human consumption commercialized in Spain. Food Microbiol..

[14] Andrade G.C.R.M., Pimpinato R.F., Francisco J.G., Monteiro S.H., Calori-Domingues M.A., Tornisielo V.L. (2018). Evaluation of mycotoxins and their estimated daily intake in popcorn and cornflakes using LC-MS techniques. LWT.

[15] Mir S.A., Dar B.N., Shah M.A., Sofi S.A., Hamdani A.M., Oliveira C.A., Moosavi M.H., Khaneghah A.M., Sant’Ana A.S. (2021). Application of new technologies in decontamination of mycotoxins in cereal grains: Challenges, and perspectives. Food Chem. Toxicol..

[16] Xue W., Macleod J., Blaxland J. (2023). The use of ozone technology to control microorganism growth, enhance food safety and extend shelf life: A promising food decontamination technology. Foods.

[17] Korzec D., Freund F., Bäuml C., Penzkofer P., Nettesheim S. (2024). Hybrid Dielectric Barrier Discharge Reactor: Characterization for Ozone Production. Plasma.

[18] Uzoma S., de Alencar E.R., Faroni L.R.D.A., de Assis Silva M.V., Sitoe E.D.P.E., Pandiselvam R., Machado S.G. (2024). Association between low-temperature drying and ozonation processes to control pests and preserve maize quality. Food Control.

[19] Alencar E.R., Faroni L.R.D.A., Soares N.D.F.F., Silva W.A., Carvalho M.C.S. (2012). Efficacy of ozone as a fungicidal and detoxifying agent of aflatoxins in peanuts. J. Sci. Food Agric..

[20] Savi G.D., Gomes T., Canever S.B., Feltrin A.C., Piacentini K.C., Scussel R., Oliveira D., Machado-de-Ávila R.A., Cargnin M., Angioletto E. (2020). Application of ozone on rice storage: A mathematical modeling of the ozone spread, effects in the decontamination of filamentous fungi and quality attributes. J. Stored Prod. Res..

[21] Oliveira J.M., Alencar E.R., Blum L.E.B., Ferreira W.F.S., Botelho S.D.C.C., Racanicci A.M.C., Leandro E.S., Mendonça M.A., Moscon E.S., Bizerra L.V.A.S. (2020). Ozonation of Brazil nuts: Decomposition kinetics, control of *Aspergillus flavus* and the effect on color and on raw oil quality. LWT.

[22] Mendez F., Maier D.E., Mason L.J., Woloshuk C.P. (2003). Penetration of ozone into columns of stored grains and effects on chemical composition and processing performance. J. Stored Prod. Res..

[23] Hardin J.A., Jones C.L., Bonjour E.L., Noyes R.T., Beeby R.L., Eltiste D.A., Decker S. (2010). Ozone fumigation of stored grain; closed-loop recirculation and the rate of ozone consumption. J. Stored Prod. Res..

[24] Silva M.V.A., Faroni L.R.A., Martins M.A., Sousa A.H., Bustos-Vanegas J.D. (2020). CFD simulation of ozone gas flow for controlling *Sitophilus zeamais* in rice grains. J. Stored Prod. Res..

[25] (1991). Eurocode 1: Actions on Structures: Part 1-7: General Actions-Accidental Actions.

[26] Mehta D., Hawley M.C. (1969). Wall effect in packed columns. Ind. Eng. Chem. Process Des. Dev..

[27] Cohen Y., Metzner A.B. (1981). Wall effects in laminar flow of fluids through packed beds. AIChE J..

[28] Karababa E. (2006). Physical properties of popcorn kernels. J. Food Eng..

[29] Rakness K., Gordon G., Langlais B., Masschelein W., Matsumoto N., Richard Y., Robson C.M., Somiya I. (1996). Guideline for measurement of ozone concentration in the process gas from an ozone generator. Ozone Sci. Eng..

[30] Weber É.A. (2005). Exelência em Beneficiamento e Armazenagem de Grãos.

[31] Kaliyan N., Morey R.V., Wilcke W.F., Carrillo M.A., Cannon C.A. (2007). Low-temperature aeration to control Indianmeal moth, Plodia interpunctella (Hübner), in stored grain in twelve locations in the United States: A simulation study. J. Stored Prod. Res..

[32] Olatunde G., Atungulu G.G., Sadaka S. (2016). CFD modeling of air flow distribution in rice bin storage system with different grain mass configurations. Biosyst. Eng..

[33] Jayas D.S., White N.D. (2003). Storage and drying of grain in Canada: Low-cost approaches. Food Control.

[34] Van Leeuwen J. (2015). Proposed OS&E requirement: Measuring ozone dosage. Ozone Sci. Eng..

[35] Venegas J.G., Harris R.S., Simon B.A. (1998). A comprehensive equation for the pulmonary pressure-volume curve. J. Appl. Physiol..

[36] Pitt J.I., Hocking A.D. (2009). Fungi and Food Spoilage.

[37] Pitt J.I., Hocking A.D., Glenn D.R. (1983). An improved medium for the detection of *Aspergillus flavus* and *A. parasiticus*. J. Appl. Bacteriol..

[38] Silva M.V.D.A., Faroni L.R.D.A., Alencar E.R., Sousa A.H., Cecon P.R., Nogueira J.V.F., Mason Filho V. (2022). Ozone Injection at Low Pressure: Decomposition Kinetics, Control of *Sitophilus zeamais*, and Popcorn Kernel Quality. Ozone Sci. Eng..

[39] García-Pinilla S., Gutiérrez-López G.F., Hernández-Sánchez H., Cáez-Ramírez G., García-Armenta E., Alamilla-Beltrán L. (2021). Quality parameters and morphometric characterization of hot-air popcorn as related to moisture content. Dry. Technol..

[40] ASAE-American Society of Agricultural Engineers (2000). Moisture Measurement-Unground Grain and Seeds.

[41] Vieira R.D., Tekrony D.M., Egli D.B., Rucker M. (2001). Electrical conductivity of soybean seeds after storage in several environments. Seed Sci. Technol..

[42] Systat Software (2011). SigmaPlot for Windows, Version 12.0..

[43] Silva M.V.A., Faroni L.R.A., Sousa A.H., Prates L.H.F., Abreu A.O. (2019). Kinetics of the ozone gas reaction in popcorn kernels. J. Stored Prod. Res..

[44] McKenzie K.S., Sarr A.B., Mayura K., Bailey R.H., Miller D.R., Rogers T.D., Norred W.P., Voss K.A., Plattner R.D., Kubena L.F. (1997). Oxidative degradation and detoxification of mycotoxins using a novel source of ozone. Food Chem. Toxicol..

[45] Rositano J., Nicholson B.C., Pieronne P. (1998). Destruction of cyanobacterial toxins by ozone. Ozone Sci. Eng..

[46] Parsons S. (2004). Advanced Oxidation Processes for Water and Wastewater Treatment.

[47] Ferreira W.F.D.S., Alencar E.R., Blum L.E.B., Ferreira M.D.A., Mendonca M.A., Racanicci A.M.C., Urruchi W.M.I. (2021). Ozonation of Brazil nuts in aqueous media at different ph levels: Ozone decomposition, *Aspergillus flavus* inactivation, and effects on nut color and crude oil lipid profile. Ozone Sci. Eng..

[48] Wu J., Doan H., Cuenca M.A. (2006). Investigation of gaseous ozone as an anti-fungal fumigant for stored wheat. J. Chem. Technol. Biotechnol. Int. Res. Process Environ. Clean Technol..

[49] Luz S.R., Villanova F.A., Rockembach C.T., Ferreira C.D., Dallagnol L.J., Monks J.L.F., Oliveira M. (2022). Reduced of mycotoxin levels in parboiled rice by using ozone and its effects on technological and chemical properties. Food Chem..

[50] Wen G., Liang Z., Xu X., Cao R., Wan Q., Ji G., Lin W., Wang J., Yang J., Huang T. (2020). Inactivation of fungal spores in water using ozone: Kinetics, influencing factors and mechanisms. Water Res..

[51] Liang Z., Xu X., Cao R., Wan Q., Xu H., Wang J., Lin Y., Huang T., Wen G. (2021). Synergistic effect of ozone and chlorine on inactivating fungal spores: Influencing factors and mechanisms. J. Hazard. Mater..

[52] Gökmen S. (2004). Effects of moisture content and popping method on popping characteristics of popcorn. J. Food Eng..

[53] Pohndorf R.S., Lang G.H., Ferreira C.D., Ziegler V., Goebel J.T., Oliveira M. (2019). Kinetic evaluation and optimization of red popcorn grain drying: Influence of the temperature and air velocity on the expansion properties and β-carotene content. J. Food Process Eng..

[54] MAPA Normative Instruction Nº 61. Brazilian National Ministry of Agriculture, Livestock, and Supply MAPA (22 December 2011). https://sistemasweb.agricultura.gov.br/sislegis/action/detalhaAto.do?method=visualizarAtoPortalMapa&chave=263800632.

[55] Sweley J.C., Rose D.J., Jackson D.S. (2013). Quality traits and popping performance considerations for popcorn (*Zea mays everta*). Food Rev. Int..

[56] Incropera F.P., DeWitt D.P., Bergman T.L., Lavine A.S. (1996). Fundamentals of Heat and Mass Transfer.

[57] Uddin Z., Suppakul P., Boonsupthip W. (2016). Effect of air temperature and velocity on moisture diffusivity in relation to physical and sensory quality of dried pumpkin seeds. Dry. Technol..

[58] Fessel A.S., Vieira R.D., Cruz M.C.P.D., Paula R.C.D., Panobianco M. (2006). Electrical conductivity testing of corn seeds as influenced by temperature and period of storage. Pesqui. Agropecu. Bras..

[59] Paraginski R.T., Souza N.L., Alves G.H., Ziegler V., Oliveira M., Elias M.C. (2016). Sensory and nutritional evaluation of popcorn kernels with yellow, white and red pericarps expanded in different ways. J. Cereal Sci..

[60] Cañizares L.D.C.C., Silva Timm N., Ramos A.H., Neutzling H.P., Ferreira C.D., Oliveira M. (2020). Effects of moisture content and expansion method on the technological and sensory properties of white popcorn. Int. J. Gastron. Food Sci..

[61] Marston K., Khouryieh H., Aramouni F. (2015). Evaluation of sorghum flour functionality and quality characteristics of gluten-free bread and cake as influenced by ozone treatment. Food Sci. Technol. Int..

[62] Zhu F. (2018). Effect of ozone treatment on the quality of grain products. Food Chem..

